# Data on a rat infection model to assess porous titanium implant coatings

**DOI:** 10.1016/j.dib.2018.10.157

**Published:** 2018-11-02

**Authors:** M. Croes, H. de Visser, B.P. Meij, K. Lietart, B.C.H. van der Wal, H.C. Vogely, A.C. Fluit, C.H.E. Boel, J. Alblas, H. Weinans, S. Amin Yavari

**Affiliations:** a Department of Orthopedics, University Medical Center Utrecht, Utrecht, the Netherlands; b Department of Clinical Sciences of Companion Animals, Faculty of Veterinary Medicine, Utrecht University, Utrecht, the Netherlands; c 3D Systems - LayerWise NV, Leuven, Belgium; d Department of Metallurgy and Materials Engineering, KU Leuven, Leuven, Belgium; e Department of Medical Microbiology, University Medical Center Utrecht, Utrecht, the Netherlands; f Department of Biomechanical Engineering, Delft University of Technology, Delft, the Netherlands; g Department of Rheumatology, University Medical Center Utrecht, Utrecht, the Netherlands

## Abstract

A model is needed to study the effectiveness of different anti-bacterial coatings on complex metal implants in a bone environment. This article shares data on the design of porous titanium implants for intramedullary implantation in the proximal rat tibia. The implant length, diameter and porosity were optimized after testing on cadaveric specimens. This article shares data on which parameters are critical to establish a chronic implant infection in Sprague Dawley rats when using the new implant design. To this end, different strains of *Staphylococcus aureus* and inoculation doses were investigated.

**Specifications table**

**Table:** 

Subject area	Biology, materials engineering
More specific subject area	Biology: microbiology, animal models, osteomyelitis
	Materials engineering, orthopedic implants, porous titanium, anti-bacterial coating
Type of data	Images (microCT, scanning electron microscopy), histogram (colony forming units).
How data were acquired	microCT (Quantum FX; PerkinElmer)
Data format	Analyzed CFU data and Raw images.
Experimental factors	Porous titanium implants were implanted in the proximal rat tibia following inoculation with *Staphylococcus aureus* (*S. aureus*). The infection rate and bone changes were evaluated after 28 days.
Experimental features	Porous titanium implants were produced by direct metal printing technology.
	Bacteria were cultured to mid-log phase and used at different doses.
	The intramedullary canal was accessed through a hole drilled in the proximal tibia. The canal was inoculated with S. *aureus*, followed by placement of the implant.
	Infection was quantified in terms of the number of colony-forming units (CFU) after homogenization of the bones or after sonication of the implants. The bone changes were measured by microCT imaging. The bodyweight was also monitored during the study.
Data source location	Department of Orthopedics, University Medical Center Utrecht, The Netherlands.
Data accessibility	Data are with this article.
Related research article	Antibacterial and immunogenic behavior of silver coating on additively manufactured porous titanium [1].

**Value of the data**

• The data can be adopted by researchers to design their *in vivo* experiments to test the effectiveness of anti-bacterial coatings on complex metal implants. The implant type, the rat breed, the *S. aureus* strain and the bacterial inoculation dose have been considered in this study.
• The data can be used by researchers to develop novel cost-effective and clinically relevant model to study the anti-bacterial properties of new implant coatings.

## 1 Data

This article shares data on the design of porous titanium implants for intramedullary implantation in the proximal rat tibia. In addition, the data of which parameters are critical to establish chronic infection in conjunction with porous titanium implants are shared with this data article.

### 1.1 Implant design

**Fig. 1:**
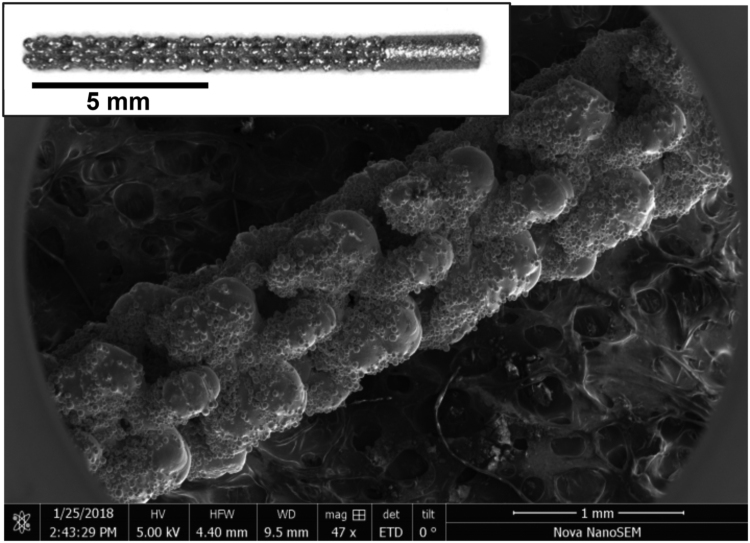
A 47-fold magnification scanning electron microscopy image of the porous implant, designed for implantation in the proximal rat tibia. The macroscopic image is shown in the inset.

**Fig. 2:**
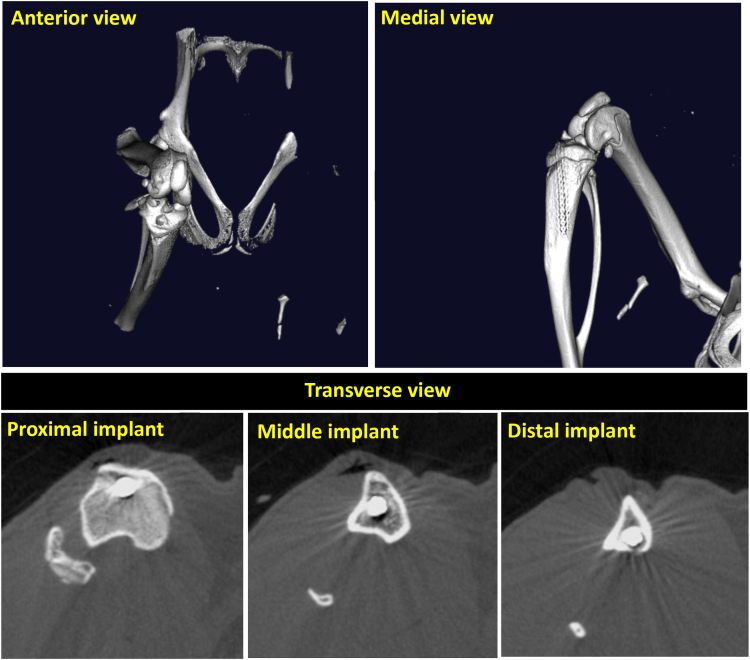
MicroCT images showing the implant placement (upper panels) and the implant position relative to the cortical bone (lower panel).

By performing cadaveric tests with different rod-shape implant designs (i.e. diameter, length, porosity), it was found that an implant diameter of 1.1 mm and a total length of 15 mm was suitable for implantation into the proximal tibia of adult male rats, when accessing the intramedullary canal from the stifle joint as described before [2]. Larger implants were either associated with fracture of the bone, or limited the insertion of the implant into at least a third of the tibial length. These implant dimensions resulted in a maximum porosity of 50 ± 5%, and a minimum pore size of 216 ± 73 μm. The strut thickness was 224 ± 71 μm. A 3 mm solid portion was incorporated as the proximal part of the implants to prevent bleeding or leakage of biomolecules from the medullary cavity into the stifle joint. The final implant design is shown in Fig. 1. The implant placement *in vivo* is shown in Fig. 2.

### 1.2 *in vivo* infection parameters

No osteomyelitis-associated bone changes were observed in the PBS control group. All groups treated with *S. aureus* demonstrated periosteal new bone formation and mild cortical thickening. The group receiving a pre-established implant and the group receiving 10^6^ CFU *S. aureus* strain ATCC 49230 showed most prominent osteolysis of cortical bone. The degree of osteolysis was variable in the other groups.

We defined chronic osteomyelitis as the presence of bone and implant infection after 4 week evaluation to allow comparison with other reported osteomyelitis models [3], [4]. At day 28 (Fig. 4), bacteria were cultured from one of the bones that had been inoculated with PBS. Considering the relative low CFU and the absence of any bone changes in this specimen, the low-grade infection was likely the result of surgical contamination. Similar to the positive control implant with pre-attached bacteria, the peri-operative injection of 10^6^ CFU of strain ATCC 49230 resulted in bone and implant infection in all animals. CFU assessment on contralateral tibiae not receiving implant or bacterial inoculation never demonstrated colony formation. There was no apparent correlation between the CFU at day 28 and a change in bodyweight (not shown).

## 2 Experimental design, materials and methods

### 2.1 Experimental design

Porous titanium implants were produced by direct metal printing (DMP) as previously described [1]. The implant length, diameter and porosity were optimized by cadaveric testing. Using these implants, it was determined which *S. aureus* strain and dose was needed to induce reproducible implant-associated infection. As a negative control, one group received PBS inoculation together with an implant. As a positive control, one group received an implant with pre-colonized bacteria. In all animals, only the left tibia received an implant, either or not with bacterial inoculation in the intramedullary cavity, while the right tibia served as control. The virulence of the *S. aureus* strains ATCC 6538 and ATCC 49230 were compared. Strain ATCC 6538, originally isolated from a human lesion, is commonly used to test antimicrobials. This strain is known to produce biofilm on titanium implants *in vitro* [5], but its ability to cause chronic infection in the rat tibia is not yet established. Strain ATCC 49230 was originally isolated from a patient with chronic osteomyelitis, and was already shown to cause implant-infections in the rat tibia in combination with an intramedullary k-wire [2]. However, its virulence in combination with our specific implant design (i.e. porous structure and only extending the proximal tibia) was unknown. The following groups were investigated in a total of 28 rats: PBS (*n* = 4), 10^3^ CFU ATCC 6538 (*n* = 4), 10^4^ CFU ATCC 6538 (*n* = 4), 10^5^ CFU ATCC 6538 (*n* = 4), 10^4^ CFU ATCC 49230 (*n* = 4), 10^6^ CFU ATCC 49230 (*n* = 4), pre-colonized ATCC 49230 (*n* = 4).

### 2.2 Bacterial culture

*S. aureus* (ATCC 6538 or ATCC 49230, Manassas, VA) were used as pathogen and grown as previously described [1]. The bacterial suspensions were extensively washed with PBS and diluted to a concentration of 1 × 10^3^–1 × 10^6^ CFU/10 μl PBS for *in vivo* injection. As a positive control, implants were made with pre-colonized bacteria, using a similar protocol that has been reported before for stainless steel rods [6]. A fresh bacterial culture was diluted in TSP +1% (w/v) glucose to OD = 0.001 (approximately 5 × 10^5^ CFU/mL). The rods were placed in this bacterial suspension overnight at 37 °C on a shaker. By sonication of the implants and plate counting, it was found that the implants were pre-colonized with 1.5 × 10^6^ CFU/implant on average.

### 2.3 Animal model

The animal experiments were performed after approval of the local Ethics Committee for Animal Experimentation (Utrecht University, The Netherlands) and the Central Authority for Scientific Procedures on Animals (approved protocol AVD115002016445). Male Sprague Dawley rats (14-week old, Charles River, L’Arbresle, France) were housed at the Central Laboratory Animal Institute (Utrecht University). Food and water were available *ad libitum*. Surgery was performed under general anesthesia with 2–3% isoflurane. The animals were given Buprenorphine (0.03 mg/kg s.c.; Temgesic®, RB Pharmaceuticals Limited, Slough, UK) for pain relief once pre-operatively and Carprofen (4 mg/kg s.c., Rymadil®, Pfizer Animal Health, Capelle a/d Ijssel, The Netherlands) once peri-operatively and twice post-operatively at 24 h intervals. The left hind leg was shaved and disinfected with povidone-iodine. A 2 cm para-patellar incision was made to open the skin and fascia. The patella tendon was dissected from the muscle tissue along its lateral side and dislocated medially. An opening was created in the tibial canal using a hand drill with 1.2 mm Ø steel Kirschner wire (Depuy Synthes, Raynham, MA). A microsyringe (Hamilton, Reno, NV) was used to inject 10 μl of PBS either or not containing *S. aureus* halfway into the medullary cavity. The implant was immediately inserted and the patella tendon was re-attached with sutures (PDS II®, Ethicon, Somerville, NJ). The skin and fascia were closed with a suture (Monocryl®, Ethicon). The bodyweight was monitored regularly. The rats were euthanized with CO_2_ after 28 days.

### 2.4 Determination of bacterial load

Bones were weighed and homogenized (Polytron PT3100; Kinetica Benelux, Best, The Netherlands) under sterile conditions. Serial dilutions were cultured on blood agar plates in triplicate. CFU were counted and normalized to the weight of the fragments. The implants were rinsed three times and sonicated for 1 min in PBS. Subsequently, a sample was taken for CFU determination by plate counting. Other data show that the CFU counts established with this method correlate with the bacterial burden as shown by scanning electron microscopy (SEM) [1].

### 2.5 MicroCT imaging

**Fig. 3:**
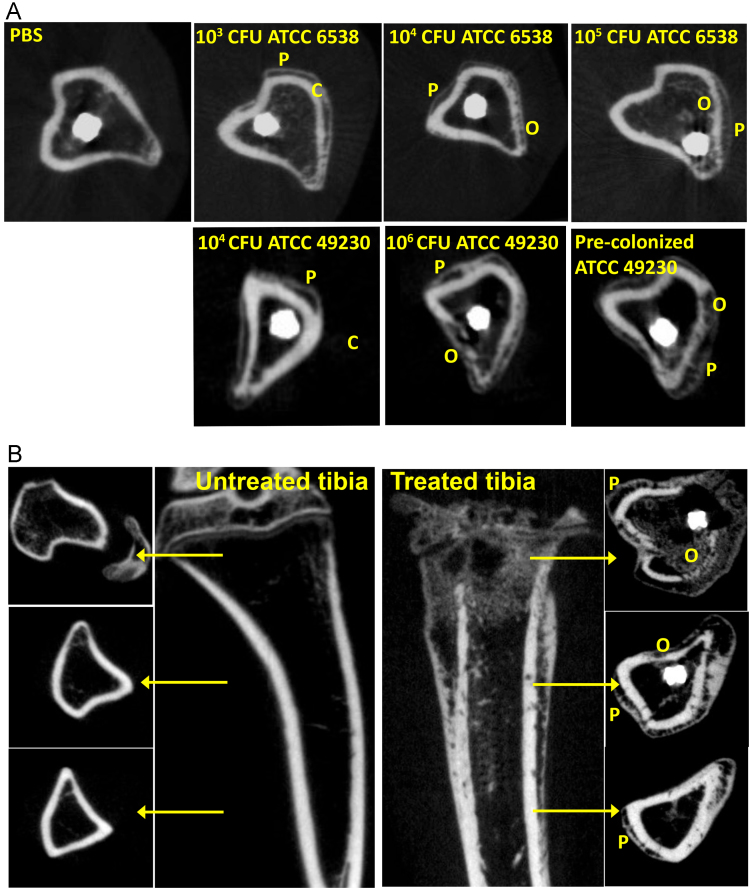
MicroCT images showing the bone morphology after 28 days. (A) Representative transversal images taken from a left tibia receiving implant, either with (ATCC 6538 or ATCC 49230) or without (PBS) bacterial inoculation in the medullary cavity. (B) Representative frontal and transversal images of a right tibia not receiving implant (untreated) and a left tibia receiving implant and inoculation with 10^6^ CFU *S. aureus* ATCC 49230 in the intramedullary cavity (treated). Osteolysis (O) and periosteal new bone formation (N) were indicative of osteomyelitis.

MicroCT (Quantum FX; PerkinElmer, Waltham, MA) images were acquired with a tube voltage of 90 kV, a tube current of 180 mA, and a field of view of 30 mm. The images were represented as a stack of 2D TIFF images with a resolution of 60 μm. Analyses were performed with the BoneJ plugin (version 1.3.12) in ImageJ freeware version 1.48 (U.S. National Institutes of Health). The bone changes were evaluated qualitatively by microCT for the presence of periosteal new bone formation, cortical thickening, and osteolysis (Fig. 3), as these bone changes are commonly seen in patients with osteomyelitis [7]. In other studies by our group, the microCT bone changes were found to correlate with increased monocyte/macrophage and lymphocyte infiltrations around the implant, and increased osteoclasts in the bone by histopathological examination [1].

**Fig. 4:**
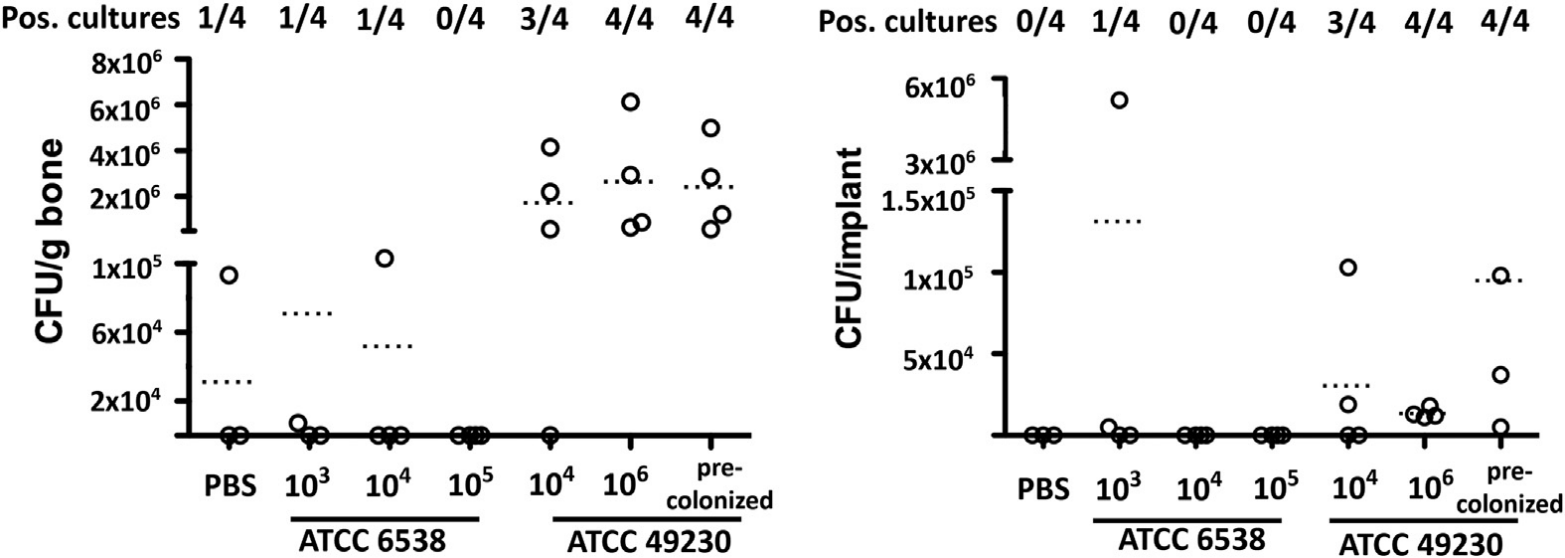
Presence of infection at day 28. Different *S. aureus* strain and inoculation doses were tested in conjunction with the porous titanium implant in the rat tibia. Bone and implant infection was evaluated following contamination with PBS, *S. aureus* strain ATCC 6538, or *S. aureus* strain 49230. Presence of infection is represented by the mean colony-forming-unit (CFU) count and the number of culture-positive samples in each group.
